# Piloting a research mentorship programme in a low-resource setting in Zimbabwe

**DOI:** 10.4102/jphia.v16i1.868

**Published:** 2025-05-12

**Authors:** Danai T. Zhou, Celia M.J. Matyanga, Munyaradzi Madhombiro, Vinie Kouamou, Precious K. Hove, Sarudzai Muyambo, Elizabeth Gori, Fortunate Farirai, Betty Mukuwapasi, Taona E. Mudhluli, Getrude D. Gwenzi, Enetia D. Bobo, Jenipher Chigerwe, Justin Chirima, Ratidzo Chirimo, Tonny P. Tauro, Mellisa B. Sagandira, Winnie Y. Mozirandi, Natsayi Chiwaye, Hardlife Rambwawasvika, Violet P. Dudu, Winnet E. Chipato, Yvonne O. Nyararai, Faith W. Kadzviti, Nomagugu Ndlovu, Upenyu N. Mupfiga, Hardlife Muhoyi, Runyararo Mano

**Affiliations:** 1 Department of Laboratory Diagnostic and Investigative Sciences, Faculty of Medicine and Health Sciences, University of Zimbabwe, Harare, Zimbabwe; 2 Department of Pharmacy and Pharmaceutical Sciences, Faculty of Medicine and Health Sciences, University of Zimbabwe, Harare, Zimbabwe; 3 Department of Psychiatry, Faculty of Medicine and Health Sciences, University of Zimbabwe, Harare, Zimbabwe; 4 Department of Medicine, Faculty of Medicine and Health Sciences, University of Zimbabwe, Harare, Zimbabwe; 5 Department of Business, Faculty of Arts, Marondera University of Agricultural Science and Technology, Marondera, Zimbabwe; 6 Department of Biotechnology, Faculty of Science, Bindura University of Science Education, Bindura, Zimbabwe; 7 Department of Basic Sciences, Faculty of Veterinary Sciences, University of Zimbabwe, Harare, Zimbabwe; 8 Organisation of Women in Science for the Developing World, Zimbabwe National Chapter, Harare, Zimbabwe; 9 Department of Soil Sciences, Faculty of Agriculture, North-West University, Potchefstroom, South Africa; 10 Department of Biochemistry, Faculty of Medicine and Health Science, Midlands State University, Gweru, Zimbabwe; 11 Department of Social Sciences, Faculty of Humanities, University of Johannesburg, Johannesburg, South Africa; 12 Department of Ecological Science, Faculty of Agriculture, Bindura University of Science Education, Bindura, Zimbabwe; 13 Department of Fuels and Energy, Faculty of Engineering, Midlands State University, Gweru, Zimbabwe; 14 Department of Mathematics and Computer Science, Great Zimbabwe University, Masvingo, Zimbabwe; 15 Department of Soil Science and Productivity, Faculty of Agriculture, Marondera University of Agricultural Science and Technology, Marondera, Zimbabwe; 16 Department of Biotechnology, Faculty of Science, Midlands State University, Gweru, Zimbabwe; 17 Department of Biotechnology, Faculty of Science, Chinhoyi University of Technology, Chinhoyi, Zimbabwe; 18 Department of Chemical and Metallurgical Engineering, Faculty of Engineering, School of Chemical and Metallurgical Engineering University of the Witwatersrand, Johannesburg, South Africa; 19 Department of Soil Science, Zimbabwe Sugar Association Experiment Station, Chiredzi, Zimbabwe; 20 Department of Agricultural Economics, Faculty of Agriculture and Environmental Sciences, Bindura University of Science Education, Bindura, Zimbabwe; 21 Department of Pharmaceutical Technology, Faculty of Pharmacy, Harare Institute of Technology, Harare, Zimbabwe; 22 Department of Biology/Biochemistry, Faculty of Applied Science, National University of Science and Technology, Bulawayo, Zimbabwe; 23 Department of Food Science and Nutrition, Faculty of Science, Midlands State University, Gweru, Zimbabwe; 24 Department of Enviromental Sciences, Faculty of Geography, Midlands State University, Gweru, Zimbabwe; 25 Department of Geography Geospatial Sciences and Earth Observation, Faculty of Science, University of Zimbabwe, Harare, Zimbabwe; 26 Department of Paediatrics, Faculty of Medicine and Veterinary Sciences, University of Namibia, Windhoek, Namibia

**Keywords:** Africa, gender, mentorship, pilot, project, research, STEMM

## Abstract

**Background:**

Women continue to be underrepresented in science, technology, engineering, mathematics and medicine (STEMM), globally including in Africa and, indeed in Zimbabwe. The gender gap, absence of formal research mentorship and the male-dominated academic culture common among low- and middle-income countries makes scientific growth dire for Africa- and Zimbabwe-based female science researchers.

**Aim:**

To address some of these challenges, a group of researchers (90% female) created the African Excellence in Research Initiative (AFRIESEARCHI) Zimbabwe Gender in STEMM Mentorship Programme.

**Setting:**

Public universities and research institutions in Zimbabwe.

**Methods:**

The team crafted a research mentorship curriculum, informed by stakeholder engagement and needs assessment, and piloted it from October 2021 to December 2022.

**Results:**

The inaugural 12-month programme capacitated 30 members (80% female) with skills for research. The participants’ mean age was 42.5 (6.9) years, with minimum qualifications of Master’s degrees. Specifically, 5 (17%) members either registered for or graduated with doctoral degrees, 14 (50%) members completed visiting fellowships. Five individual projects were awarded grants all totalling over $300 000.00, while this mentorship project was shortlisted for the Free STEM Fund award (€50 000.00) for the 2022–2023 cycle. Half of shortlisted team members were selected for the competitive Zimbabwean Emerging Faculty Development Program. Almost 90% of participants were satisfied with their mentorship experience, although resources and time were needed.

**Conclusion:**

Despite challenges, the team resolved the need to prioritise formalised research mentorship, within the Zimbabwe setting.

**Contribution:**

Such efforts will enhance scientific growth for women (and indeed all academic researchers) in the sciences.

## Introduction

Research mentorship is critical for advancing science, but there are scarce practical approaches for promoting it in resource-limited settings.^1^ The 2022 World Economic Forum reports that women continue to be underrepresented in Science, Technology, Engineering, Mathematics and Medicine (STEMM) fields.^2^ This is exaggerated in Africa where completion of education by women remains among the lowest.^3^ In Zimbabwe, numbers of women in STEMM are also diminished, while many are leaving STEMM at all stages of the career pipeline.^4^ In addition, there are challenges of funding for researchers in low- and middle-income countries (LMICs) such as Zimbabwe.^5^ The low access to research funding^5^ and gender imbalance in STEMM,^2^ are both because of multiple reasons. These include: limited local funding towards research, low resources placed towards mentorship, insufficient knowledge about funding application, time constraints because of work or life responsibilities, a lack of role models who have won international research grants and few mentors to guide the grant application process.^6^

Because of a dearth of research mentorship and research funding constraints,^1,5,7^ researchers and faculty members in Zimbabwean universities and research institutions, are struggling with the fulfilment of the requirements of the new Education 5.0 strategy. Education 5.0 is the major thrust of Zimbabwe’s Higher Education Ministry, which seeks to encourage the development of ‘doers, thinkers, collaborators and problem-solvers’.^8^ All the five prongs of the Education 5.0 approach, which are teaching, research, innovation, industrialisation and community service,^9^ together with gender parity, are key drivers for United Nations (UN) sustainable development goals. To address some of the above challenges, a gender-sensitive research mentorship programme was developed and piloted by 10 early- to mid-career researchers from Zimbabwe (90% female).

### Problem statement

Mentorship is a proven method, essential in developing science professionals for the 21st century.^10,11^ Although transformative for both mentors and mentees, mentorship rarely receives the focused attention, material and financial support as other aspects of professional development, in low-resource settings.^12,13^ There is an urgent need to formalise and normalise research mentorship, in order to increase research funding access, mainstream innovation and enhance scientific excellence in many LMICs, such as Zimbabwe.^14,15^

### Project aim

The aim of this capacity-building programme is to strengthen research mentorship by developing an excellent, gender-sensitive, collaborative research culture, for female scientists and researchers, for Zimbabwe’s research hubs and institutions of higher learning.

### The theory of change

**FIGURE 1:**
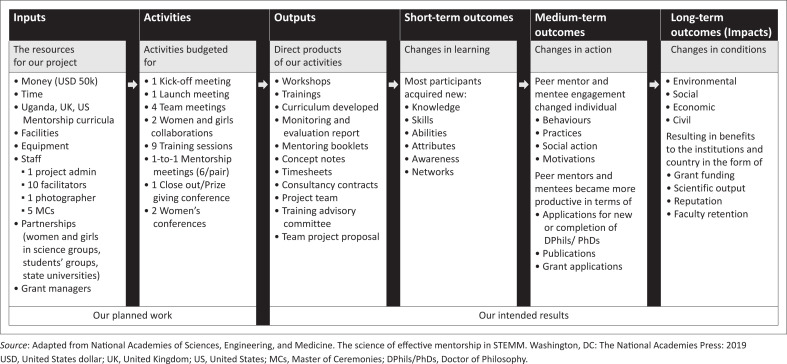
The Project Logic Model showing the components of planned work and intended results.

A theory of change (TOC) provided a framework for our programme’s activities and outputs, all designed to lead to the desired outcomes and impact. The TOC was driven by the observation that researchers from low-resource settings face significant barriers to securing funding, publishing research and translating findings into policy and practice. Hence, our programme’s long-term goal is to strengthen the capacity of researchers from a low-resource setting to secure funding, and produce high-quality research. Our programme’s intermediate outcomes are for researchers to: demonstrate increased knowledge and skills in writing competitive grant proposals; publish more research articles in reputable journals; and produce high-quality research that meets international standards. Guided by the TOC, our short-term outcomes are that researchers should: (1) express increased confidence in their ability to secure funding and conduct research; (2) develop clear research plans and proposals; and (3) establish relationships with experienced mentors and peer mentees. Our programme outputs are: (1) an established, structured mentorship programme, providing training and support to researchers; (2) trained researchers on grantsmanship, research design and methods; (3) developed and refined research proposals with mentor guidance and (4) peer reviewed draft research proposals and draft articles. Our programme assumptions are that: researchers are motivated to improve their grantsmanship and research skills and mentors have the necessary expertise and experience to provide effective guidance (see Figure 1^16^ for the logic model).

### Intervention packages

An intervention package was designed to provide a comprehensive support system for mentees, addressing their research needs, skills and knowledge. By providing mentor training, mentee support, regular meetings and feedback, resource support, and monitoring and evaluation (M&E), this package’s aim was to enhance the effectiveness of the mentorship programme.

## Research methods and design

### Setting up the team and developing a training curriculum

The principal investigator (PI) and co-PIs co-designed the initiative entitled the ‘African Excellence in Research Initiative (AFRIESEARCHI) Zimbabwe Gender in Science, Technology, Engineering, Mathematics, and Medicine (STEMM) Program’ aka ‘AFRIESEARCHI Zim GIS Program’. They then coalesced a multidisciplinary core team of 10 peer mentors. The peer mentors comprised one PI (postdoc), two co-PIs (one postdoc and one Doctor of Philosophy [DPhil] candidate) and seven members, each having at least a Masters’ degree in any STEMM field, a publication and a history of research funding. One programme administrator and 20 mentees were competitively selected by the peer mentors, from a pool of 49 early-career researchers, with at least a Masters’ degree, and at least one published article.

The peer mentors developed the mentorship curriculum (Table 1), based on a general needs assessment from published and grey literature, and a targeted needs assessment achieved by surveying team members. In addition, the curriculum development was informed by similar programmes from Africa (Uganda, Zimbabwe),^12,13,14,15^ the United Kingdom (UK) (https://bit.ly/3PhWTk8) and the United States (US),^16,17,18,19^ then adapted for the local setting. Based on the needs assessment, the training for both peer mentors and mentees covered a range of topics such as: mentorship versus supervision, effective mentorship, gender in STEMM, work–life balance, sexual harassment, leadership styles, networking, proposal writing, grant writing, responsible conduct of research and publishing.

**TABLE 1:** Curriculum and programme toolbox developed for the African Excellence in Research Initiative ZIM GIS mentorship project.

Module	Mentors	Mentors and mentees
1.	Team building, research collaboration, work-life balance, health and fitness	Introduction to the African Excellence in Research Initiative: The Zimbabwe Gender in STEMM Project
2.	Criteria for selection of trainees and developing enrolment forms, monitoring and evaluation	The effective mentorship relationship: Mentoring models and techniques
3.	Curriculum development: From general and targeted needs assessment to implementation and evaluation, curriculum content delivery, formative and summative evaluation	Handling mentee–mentor relationships: The mentorship relationship and stages of mentorship.
4.	Gender in STEMM: What are the issues? Sexual harassment and Gender based violence	Gender imbalance in STEMM and interventions to address it
5.	Responsible conduct of research, ethics in people, resource and data management and leadership styles	How to conduct a literature search and write the introduction, Research proposal writing: Framing the problem, Ethics and responsible conduct of research
6.	Effective communication (oral and poster, pitching, 2-min talks [2MT], writing curriculum vitae, application letters and articles) and communication for success in research using social media	Keeping one-self at the cutting edge of science, including proposal writing and publishing
7.	Successful grant writing	Basic tools for effective grant writing and resources for finding grants
8.	Keeping one-self at the cutting edge of science, including proposal writing, and publishing	Work–life balance for men and women in STEMM and effective time management tools
9.	Effective mentorship (The mentorship relationship and stages of mentorship)	Data analysis and publishing
10.	Effective mentorship (mentoring models and techniques)	Effective communication: articles, oral and poster presentations, Get Visible or Vanish: Opportunities for digital and social media in research

STEMM, Science, Technology, Engineering, Medicine and Mathematics; ZIM GIS, Zimbabwe Gender in Science.

### Programme activities

To fulfil the aims of the programme, various activities were carried out. Firstly, the project PI and co-PI raised awareness about research mentorship through authorship,^15^ and by participating in local community events (https://bit.ly/3PjStcC) and on social media (https://bit.ly/3c1zSn4). Secondly, funding applications were made to various local and global funding agencies, to support activities. Thirdly, the team then engaged a training advisory committee (TAC) comprising senior mentors and technical advisers from the University of Zimbabwe (UZ), and the University at Buffalo (UB), SUNY, US. There is significant overlap between the vision and mission of the above institutions (https://www.uz.ac.zw/ and http://www.buffalo.edu/).

To design and implement the mentorship training curriculum, the PI and co-PIs of the AFRIESEARCHI Zim GIS programme leveraged previous participation in capacity-building training. This included workshops hosted by the Africa Research Excellence Fund (AREF), UK and the UZ Faculty of Medicine and Health Sciences (FMHS). The faculty development workshops were, organised by UZ FMHS senior researchers, in collaboration with the Biomedical Research and Training Institute (BRTI), the Medical Education Partnership Initiative (MEPI) and the Health Education and Advanced Leadership program in Zimbabwe (HEALZ) among others.^15^ A programme toolbox with outcome matrix for self-assessment was adapted for this work (Table 2).^15^ A Logic Model (Figure 1) of planned work and intended results, was adapted from the National Academies of Sciences, Engineering and Medicine, 2019 and Murray S.A. et al., 2022.^16,19^

**TABLE 2:** The programme toolbox with outcome matrix.

Key research mentorship skills	Outcome matrix
Develop a comprehensive knowledge of research mentorship, including roles of mentors and mentees.	Each project team member (peer mentor or mentee) will assess their number of training and mentorship meetings attended, quality of mentorship and acquired knowledge about research mentorship, including roles of mentors and mentees.
Design a research protocol and individual development plan with clear goals or the mentorship.	Each member will assess their progress in designing research protocols for peer review, individual development plans and goals for mentorship.
Utilise the skills gained to provide mentorship to many others.	Each member will assess their confidence in providing mentorship to other people.
Demonstrate an understanding of key aspects of multidisciplinary research teams, team dynamics, responsible conduct of research and the process of scientific dissemination.	Each member will participate in multidisciplinary research team meetings and contribute positively towards building a multidisciplinary research team, demonstrating responsible conduct of research, scientific dissemination through social media and collaborative scholarship.

*Source:* Adapted from Zhou DT, Maponga CC, Madhombiro M, et al. Mentored postdoctoral training in Zimbabwe: A report on a successful collaborative effort. J Public Health Afr. 2019;10(2):a909. https://doi.org/10.4081/jphia.2019.1081

### The monitoring and evaluation plan

As per our M&E plan,^16,17,18,19^ the impact of our programme was monitored and evaluated using quarterly review reports by the study team, using captured monthly reports as a basis for assessment. At the end of each quarter, high-level evaluation meetings were held to conduct a postmortem on the status of the study focusing on strengths, weaknesses, opportunities, threats, achievements and planning.^16,17,18,19^ Key informant reports were made by responsible team members regarding number of participants attending training sessions, number of participants formally mentored, challenges experienced, remedial actions to be taken, successful strategies or processes implemented and activity outputs or outcomes that is number of feedback surveys completed, number of concept notes submitted and reviewed, number of monthly reports made and financial logs showing utilised resources.^16,17,18,19^

### Statistical analysis

For quantitative statistical analysis, we used descriptive analysis to summarise demographic characteristics, research skills and perceptions among mentees and mentors. Students’ *t*-tests at 95% confidence were used to compare continuous variables. For qualitative data, interviews and focus group discussions were transcribed verbatim and interpreted in relation to the research objectives and literature review.

### Ethical considerations

Ethical clearance to conduct this study was obtained from the Medical Research Council of Zimbabwe (No. MRCZ/A/2832).

## Results

Approximately 80% of participants are female. There is a slight difference between mentors’ and mentees’ mean age but the difference did not reach statistical significance, *p* = 0.0835. The participants’ demographics, past experience and output during or beyond the programme (from 2022 to 2025), and participants’ current roles are listed in Table 3. A total of 88.2% of participants were satisfied with their mentorship experience, although 12.8% (*n* = 4) felt the need for more resources and time (Table 4 and section ‘Some exit survey comments arranged by themes’).

**TABLE 3a:** Participants’ metadata.

Participant	Age (years)	Work experience	Level of past research exposure (number of articles)	Current role (additional grants and fellowships)
1	56	High school teacher (9 years) Junior lecturer (11 years) Senior lecturer (7 years)	M.Sc. Clin Biochem, PhD, Postdoc (8 years)	Senior lecturer Postdoc
			3 published, 3 under review	1 grant 2 research fellowships 4 visiting fellowships
2	41	Lecturer (12 years)	B.Sc. Pharmacy Hons., M.Sc. project (1 year), PhD, Postdoc (1st year)	Senior lecturer Postdoc
			6 published, 1 under review	2 grants 4 research fellowships
3	57	Clinical specialist (21 years) Lecturer (12 years)	MBChB, MMed (Psyc). PhD Masters in Epidemiology	Senior Lecturer Clinician
			1 published, 1 under review	1 grant 2 visiting fellowships
4	36	Research scientist (9 years) Lecturer (2 years)	PhD, Postdoc Virology (26 years)	Graduated with PhD in 2023, Postdoctoral fellow
			3 published, 2 under review	1 grant 2 research fellowships 1 visiting fellowship
5	49	Secondary Teacher (6 years) University Lecturer (12 years) Dean of a faculty (5 years)	PhD Business Sciences	Dean Faculty of Management and Entrepreneurial Sciences
			2 Published, 1 under review	1 grant
6	48	Lecturer	B.Sc. Hons. and M.Sc. Biotechnology projects	Lecturer Zimbabwe National Chapter Chair
			1 under review	1 grant
7	43	Lecturer	M.Sc. Biotechnology, PhD Chem. Path.	Senior Lecturer
			2 published	1 grant
8	48	Research Scientist Former OWSD National Chapter Chair	PhD Chemical Engineering 0 published	Business owner OWSD Africa Region Representative
				1 grant
9	50	High School Teacher (10 years) Lecturer (2 years)	PhD student (4th year) Neglected Plants 2 published	Lecturer PhD student
				1 grant
10	32	Technician (5 years) Lecturer (3 years)	Masters; PhD Microbiome (4th year student) 2 published	Lecturer PhD student
				2 research fellowships 2 visiting fellowships
11	52	High School Teacher (7 years) Lecturer (18 years)	M.Sc., PhD in Environmental Science	Deputy Dean
			0 published	0 fellowships
12	51	High School Teacher (8 years) Lecturer (15 years)	M.Sc., PhD Plant Biotechnology and Physiology	Senior Lecturer
			8 published	2 grants
13	35	Lecturer	PhD (Chemical Sciences) (4 years)	Graduated with PhD in 2024, Senior Lecturer
			2 published	1 grant
14	44	Researcher (8 years) Lecturer (12 years)	Masters; PhD Soil Science	Senior lecturer
			4 book chapters and 7 articles published	2 grants
15	42	Lecturer (14 years)	PhD Researcher	Senior lecturer
			9 published, 1 in print	0 grants
16	46	Paediatrician (12 years) Lecturer (7 years)	MMed, NIH D43, PhD student Paediatrics (4 years)	Lecturer, PhD student, Thesis submitted
			8 published	1 grant 1 visiting fellowship
17	43	Lecturer (14 years)	PhD student, thesis submitted (December 2024) 3 published, 2 under review	Lecturer PhD student
				2 research fellowships
18	39	Researcher (5 years) Project engineer (1 year)	Masters, PhD Chemical Engineering 2 articles	Senior lecturer Research engineer
				0 grants
19	48	Renewable energy engineer Lecturer (6 years)	Masters, work-related research	Lecturer
			0 articles	1 grant
20	42	Technician (13 years) Lecturer (3 years)	Masters in Biotech, Project Management Dip.	Lecturer
			2 articles under review	1 research fellowship
21	35	Lecturer (2 years)	M.Sc. in Applied Mathematical Modelling	Lecturer
			1 published	0 grant
22	33	Lecturer (5 years)	Masters; PhD (first year student)	Full-time PhD student
			2 published	1 grant
23	40	Project coordinator (15 years)	MPH, work-related research	Project coordinator
			3 published	0 grant
24	39	Lecturer	M.Sc. Biotechnology, PhD	Senior lecturer
			3 published	1 grant
25	51	Diagnostic lab scientist (11 years), Research lab scientist (9 years) Lecturer (3 years)	Masters; PhD (first year student) 0 published	Lecturer PhD student
				2 research fellowships 3 visiting fellowships
26	39	Lecturer (15 years) Postdoc (2 years)	PhD 3 years; Postdoc 2 years	Lecturer and Postdoc
			8 articles published so far	2 grants
27	39	Chemistry Laboratory Technician (4 years)	MPhil Soil Chemistry	PhD Student
			4 published	1 PhD scholarship
28	35	Consultancy (5 years) Lecturer (4 years)	B.Sc., M.Sc. Water Resource Management (2 years) Lecturer (4 years)	Lecturer
			10 published, 1 under review	3 grants
29	34	Lecturer	M.Sc. Chemistry	Lecturer
			0 published	0 grants
30	40	Graduate research assistant (3 years), research scientist (3 years) Lecturer (9 years)	M.Sc., PhD student (4 years)	Lecturer and PhD student
			3 published	2 grants
31	35	Project director (3 years) Postdoc (2 years) Lecturer (1 year)	PhD student Postdoc (2 years)	Project director
			0 published	1 grant

M.Sc. Clin Biochem, Master of Science Clinical Biochemistry; PhD Postdoc, Doctor of Philosophy Postdoctoral; B.Sc. Pharmacy Hons, Bachelor of Science Pharmany Honors; MBChB, Bachelor of Medicine, Bachelor of Surgery; Chem. Path., Chemical Pathology; MMed, Master of Medicine; NIH D43, National Institute of Health International Research Training Grant; Dip., diploma; MPH, Master of Public Health; MPhil, Master of Philosophy; OWSD, Organization of Women in Science for the Developing World.

**TABLE 3b:** Participants’ metadata.

Participant	Age (years)		Level of past research exposure (number of articles)	Current role (number of grants and fellowships)
	Mean	s.d.		
Total: Mentors	45.6	8.0	19 publications	11 grants and fellowships
Total: Mentees	41.0	5.9	73 publications	26 grants and research fellowships
Overall (mentors and mentees)	42.5	6.9	92 publications	37 grants and research fellowships

Note: *t*-test for mean age of mentors versus mentees; *p*-value = 0.0835.

s.d., standard deviation.

**TABLE 4:** Mid-term reflection survey results (30 peer mentors and mentees).

Question	Yes		No		Average	Range
	*n*	%	*n*	%		
1. Prior to this project, had you received any scientific career mentorship?	16	53.0	14	47.00	-	-
2. Have you received funding before?	21	70.0	9	30.00	-	-
3. If you have received funding, how many times?	-	-	-	-	2.0	1–6
4. Have you published in a peer reviewed journal?	26	87.0	4	13.00	-	-
5. If you have published in a peer reviewed journal, how many articles have you published?	-	-	-	-	8.0	1–36
6. Are you a faculty staff member?	26	87.0	4	13.00	-	-
7. Do you have research partners?	24	80.0	6	20.00	-	-
8. If yes, please specify whether they are local or international	-	-	-	-	-	-
Local	-	21.4	-			
International		-	-	78.6		
9. How many research partners or collaborators do you have currently? If none, please write none.	-	-	-	-	2.5	1–8
10. What skills were you hoping to learn when you joined the ZIM GIS project? Please list:	-	-	-	-	-	-
10.1 Article writing	5	16.7	-	-	-	-
10.2 Grant writing	13	43.3	-	-	-	-
10.3 PhD proposal writing	2	6.7	-	-	-	-
10.4 Mentoring	3	10.0	-	-	-	-
10.5 Developing confidence	1	3.3	-	-	-	-
10.6 Systematic review	2	6.7	-	-	-	-
10.7 Time management	1	3.3	-	-	-	-
10.8 Professional networking	1	3.30	-	-	-	-
10.9 Postdoc application	2	6.75	-	-	-	-
10.10 Professional development	2	3.30	-	-	-	-
10.11 Increasing research output	1	3.30	-	-	-	-
11. Have you attended all mentorship sessions?	25	83.3	5	16.7	-	-
12. If you missed a mentorship session, please state the reason/s for not attending.	-	-	-	-	-	-
Health reasons	1	-	-	-	-	-
Mentee absenteeism	3	-	-	-	-	-
13. How do you hold your mentor or mentee meetings?	-	-	-	-	-	-
Physical and virtual	4	13.3	-	-	-	-
Virtual (WhatsApp)	26	86.70	-	-	-	-
14. Were you comfortable with the mode used to hold mentor or mentee meetings?	28	93.3	2	6.70	-	-
15. Are you satisfied with your mentorship experience?	-	-	-	-	-	-
Yes	26	86.7	-	-	-	-
Maybe	-	-	4	13.30	-	-

ZIM GIS, Zimbabwe Gender in Science; PhD, Doctor of Philosophy.

### Impact of the programme’s awareness campaigns

Team members came from different institutions and various science disciplines including agriculture, biomedical sciences, chemical engineering, chemical pathology, chemistry, drug development, environmental sciences, genetics, medical virology, medicine, paediatrics, pharmacy, pharmacology, psychiatry, veterinary sciences and social sciences. Thus, the efforts of the team members and collaboration raised awareness of research mentorship at several universities, different institutions and scientific fields within Zimbabwe:

‘My approach to career networks has greatly improved. I also received some positive feedback from mentor and mentee meetings.’ (35 years old, female, mentee)

### Collaborative efforts

Beyond the lifespan of the project, the team members were invited to host a Women in Science Conference in Zimbabwe, in November 2022, while a small team of members led group mentorship sessions at the UNESCO-UZ celebration of the International Day of Women and Girls in Science, on 10 February 2023. Another opportunity was the participation of team members at the Zimbabwe Association of Medical Laboratory Sciences Students (ZAMLS) Conference on 19 March 2023. Several invitational grant writing training workshops were held in 2024 and beyond. In addition, the mentorship project was shortlisted for the Free STEM Fund award for 2023. The team is continuing to pursue funding and looks forward to continue advocating for research mentorship and women empowerment in STEMM, in Zimbabwe, as individuals, as a team and in collaboration with universities, international funding bodies and local women’s groups such as the Organization of Women in Science for the Developing World Zimbabwe National Chapter (OWSD ZNC) and groups for women academics from Zimbabwe:

‘My mentor was always available to guide, encourage and motivate me.’ (35 years old, male, mentee)

### The training advisory committee and the tiered mentorship model

**FIGURE 2:**
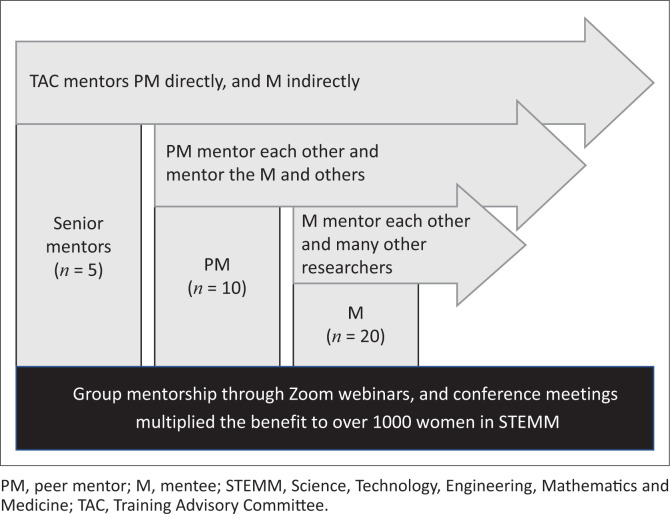
The tiered mentorship model.

The training advisory committee (TAC) members served as senior mentors, who mentored the peer mentors directly and the mentees indirectly, using a tiered model (Figure 2). Our novel tiered model, leveraged a cascading approach to knowledge transfer and support. In this structure, senior professors (senior mentors) mentored the 10 early-career researchers, who designed the programme (peer mentors), who in turn mentored two junior mentees each (*n* = 20). This tiered design allowed for efficient knowledge dissemination, as senior mentors imparted their expertise to peer mentors, who then transmitted this knowledge to their mentees. By bridging generational and experiential gaps, this model fostered a supportive community of practice, where mentors and mentees learned from and motivated one another, promoting a culture of collaborative growth and development. This model helped the peer mentors and mentees to maximise their benefit from senior mentors who are by nature very busy individuals.

### The training curriculum and programme toolbox

The training curriculum comprised 10 modules for mentors only, and 10 modules for both mentors and mentees. The modules were crafted by the team and were fluid in order to accommodate the needs of the participating team members (peer mentors and mentees). The programme toolbox with outcome matrix proved to be a necessary tool for members’ self-assessment.

### Annual plan and calendar of events

As directed by the logic model, the project implementation started with a kick-off meeting and launch, followed by a series of career development and research mentorship workshops, facilitated by external and internal team of experts. Using an andragogy approach, peer mentors were involved as internal facilitators and delivered modules alongside external facilitators. External facilitators from Zimbabwe, Africa and abroad, were invited to share experiences through a series of workshops. Peer mentors were later matched with mentees based on mentees’ needs and career goals. After 6 months of being trained, peer mentors then facilitated one-on-one mentorship for two mentees each, over a further 6 months. The online workshops and one-on-one mentorship helped team members to navigate their career transition. During the exit survey, useful comments for improvement were received from both peer mentors and mentees and arranged by themes such as mentors’ and mentees’ fields, time requirements and physical meetings and networking.

### Project impact

#### Participants’ perceived outcomes

Members of this mentorship programme reported several important achievements for example, they developed skills for scientific writing, presentation, collaborations and grant writing. Some participants formed life-long relationships and continued to mentor one another, beyond the programme lifespan.

#### Participants’ perceived outputs

Mentors and mentees reported having made tangible achievements during and after this programme. In particular, 5 (16%) members registered for or graduated with doctoral degrees, 92 articles were drafted, and submitted or published and 13 (42%) members completed regional and international visiting fellowships. Individuals were awarded postdoctoral or postgraduate or seed grants, or fellowships and 2 (7%) were selected for the highly competitive Zimbabwean Emerging Faculty Development Program (ZEFDP) by the US Embassy, Zimbabwe in partnership with the Institute of International Education (IIE). Twelve (39%) members developed competitive concept notes using the AFRIESEARCHI template, hundreds of additional people (and counting) have been formally mentored (besides those assigned in this project). The programme team co-hosted the Zimbabwean Women in Science Conference together with the OWSD ZNC and other collaborators, in 2023 and 2024. One member created a WhatsApp platform, where participating academic members and researchers share information on current grant or fellowship calls and scholarships. A more detailed list of the participants’ achievements is in Table 5.

**TABLE 5:** Participants’ achievements during and 1 year after the programme (2022–2025).

List number	Output
1	Two individuals either submitted a DPhil thesis or graduated with a DPhil, respectively.
2	Three members registered for a PhD or DPhil.
3	Ninety-two articles were drafted, and submitted or published.
4	Over 37 grant applications were awarded.
5	Two members completed visiting fellowships in Germany, three in South African institutions, one in Namibia, one in Rwanda, six travelled to the US for short-term and long-term training visits and one travelled to the UK for short-term training visits.
6	Five individual projects were awarded postdoctoral or postgraduate or seed grants, totalling over USD 300 000.00.
7	This mentorship project was shortlisted for the Free STEM Fund award (50 000 Euros).
8	Five team members acquired new jobs or consultancy or appointments..
9	Four team members were shortlisted and two were selected for the highly competitive Zimbabwean ZEFDP by the US Embassy, Zimbabwe in partnership with the IIE.
10	Twelve members developed competitive concept notes using the AFRIESEARCHI template.
11	Hundreds of additional people (and counting) have been formally mentored (besides those assigned in this project).
12	Four invitations into committees for research mentorship, women empowerment and science advocacy groups (all accepted).
13	One member submitted a proposal to host and facilitate a session at an international conference, which was accepted and successfully executed.
14	One member was promoted to senior lecturer grade and selected as an academic mentor at a state university.
15	The project team co-hosted the Zimbabwean Women in Science Conference together with the OWSD ZNC and other collaborators, in 2023 and 2024.
16	One member created a WhatsApp platform, where participating academic members and researchers share information on current grant or fellowship calls and scholarships.
17	Over 15 participants (mentors or mentees) received competitive research grants for their scientific work.

AFRIESEARCHI, African Excellence in Research Initiative; IIE, Institute of International Education; OWSD ZNC, Organization of Women in Science for the Developing World Zimbabwe National Chapter; ZEFDP, Zimbabwean Emerging Faculty Development Program; USD, United States dollar; US, United States; UK, United Kingdom; PhD/DPhil, Doctor of Philosophy; STEM, Science, Technology, Engineering, Mathematics.

#### Some exit survey comments arranged by themes

Theme 1: Peer Mentors’ and Mentees’ fields:

- Consider pairing mentor and mentee from the same field. This improves communication.
- In order to spread mentorship benefits widely future efforts should be focused by discipline and level for example engineering, vet sciences, biomedical sciences, social sciences, pharmacy, agricultural sciences, medicine, undergraduate students, postgraduate students, postdocs and so on.

Theme 2: More resources and time required (*n* = 4, 12.8%):

- Some of us had more than one goal to work on in this mentorship programme so getting to hear from other mentors and mentees progress would have been beneficial.
- I think the programme for future cohorts can be improved by increasing the duration to cater for members who have other roles to play in their work places.
- Provide more training to cover grant writing.
- Hold meetings in the late afternoons or weekends as some of us have demanding jobs and cannot attend meetings during working hours.

Theme 3: More physical or peer network meetings preferred:

- More seminars would help us learn from peer mentors and mentees.
- Holding more physical meetings would have been helpful.
- It would have been beneficial if more regular networking meetings amongst peers had been held.

Theme 4: More follow-up meetings and progress reports required:

- We would have preferred having more regular follow-up meetings.
- Sharing progress reports, for mentees, each month would have improved the experience.
- Using progress self-assessment tools, for mentees, would have give information about their goals and outputs at regular intervals for example every 3 months.

Theme 5: Positively impacted:

- The programme has been really good and I enjoyed it. I count it as a double portion because in trying to achieve my goals and objectives, I will also be fulfilling my annual work plan for my result-based management key areas of assessment at Bindura University of Science Education.
- It is also good to hear others to give their testimonies and journey in this programme and giving support to each other.
- We have grown to be a family and mechanisms should be put in place that this group will not dissolve. Thank you very much.
- The mentorship programme improved my interests in research and aspirations towards academic careers. My experience with the AFRIESEARCHI Zim GIS mentorship programme enhanced my career. I am now able to set goals regularly, challenge myself, read often, write often and be curious about my industry. The writing sessions improved my writing skills and helped me to overcome deadlines.

Additional comments:

- Institutionalisation of the mentorship will be ideal to increase its impact.
- Funding was crucial for supporting participants and more is required to ensure its sustainability.

### Perceived impact by participants

Participants responded to the question regarding their satisfaction with the mentorship experience, 88% were satisfied, although 12% were not and some felt that more time and resources would be beneficial.

**Question:** *Please comment on your mentorship experience* Some participants’ answers:

- Mentee: ‘The mentor is very helpful, always available when needed. She is helping me apply for a scholarship and is guiding me in writing a grant proposal’. (51 years old, female, lecturer)
- Mentor: ‘It’s a great opportunity to give back to others’. (56 years old, female, mentor)
- Mentor: ‘The mentees and I are in the early stages of our careers and we understand what each one is going through’. (32 years old, female, mentor)
- Mentee: ‘I have significantly improved in my writing skills. During the period, I wrote three papers. All were accepted for publication and conference presentation was accepted. I have also found a prospective PhD supervisor and we are working towards developing a research proposal’. (39 years old, male, MSc graduate)
- Mentee: ‘So far, my mentor has helped me in acquiring skills in searching for funded grants and she also gave me links to some interesting websites on short courses’. (39 years old, female, PhD student)
- Mentee: ‘My approach to career networks has greatly improved. I also received some positive feedback from mentor and mentee meetings’. (34 years old, female, lecturer)
- Mentee: ‘Each meeting has clear objectives and by the end of the meeting new tasks are set as well. This makes for progress. I have managed to send three PhD proposal [*applications*]; I am still waiting for the outcomes while at the same time I am continuing to keep applying’. (35 years old, male, lecturer)

### Group members’ achievements to date (guided by the outcome matrix)

Completion of this project culminated in peer mentors developing gender-awareness in the scientific research context, together with knowledge, attitudes and skills for collaboration, curriculum development, research mentorship, grant writing, responsible conduct of research, plus M&E. The mentees acquired skills for developing fundable project proposals, and for mentoring others, both of which will transform the Zimbabwe research and professional culture, one person at a time, with potential for wider application. Table 5 shows a list of some notable individual achievements of group members during and within 2 years of the mentorship:

‘I now understand what mentorship means.’ (32 years old, female, mentor)

## Discussion

There have been several mentorship programmes in low-resource settings. What’s unique about ours is the involvement of local participants (mostly women) in the design of the curriculum and its delivery. The project achieved its aim of contributing to local and global efforts for women empowerment in STEMM.

As the project was carried out during the coronavirus disease 2019 (COVID-19) pandemic, it helped members to maintain a sense of community, and helped to address the negative social impact of physical distancing in the context of COVID-19. It restored and maintained contact between and among Zimbabwean mentees and peer mentors, through ‘socially-distanced’ physical meetings and online webinars. This strategy was both innovative and life-changing as many researchers (and individuals) faced uncertain futures, sometimes in isolation. Many locally based researchers benefited from the online group mentorship at the start of the project, while those mentees selected into Cohort 1 received more individualised mentorship support. The project trained both peer mentors and mentees on mentorship and assisted the team members to find their own answers to several career-related challenges.

Online approaches, although common in developed countries, have great unpredictability in the African setting, where access to the internet is generally too costly and out of reach. Because of a dearth of funding opportunities and limited research support most local researchers have inadequate internet access. In spite of this unpredictability, physical distancing strategies were the norm, post the COVID-19 era, and our programme had to respond. One way we responded was to provide stipends towards transport and internet data to enable participants to travel for meetings, or access the internet, and participate in online training and group mentorship programmes virtually. The funding support was, however, minimal and team members from outside Harare, where most events were held, missed the physical meetings.

At the same time, this project provided an opportunity for both the newly trained peer mentors and more experienced senior mentors (TAC) to participate and give back, by mentoring the mentees, in a setting where mentorship, is traditionally erratic and not sustained. This initiative was indeed necessary, as there is limited support for formal mentorship programmes in the African and Zimbabwean context.

Because of a dearth of mentorship in LMIC, some supervisors purport to have dual roles of being both mentors and supervisors.^13^ However, centred on best practices, reports in literature and past experience, this project emphasised the role of a mentor that is separate from that of a supervisor, to limit conflict of interest. We initially found some resistance to the approach of separating a mentor from a supervisor, especially by those who did not fully comprehend the difference between supervision and mentorship. This controversy resonates with other low-resource setting where supervisors also ‘mentor’ their students.^13^ Despite the initial challenges, the initiative was perceived to be a timely response that brought local mentees and peer mentors together, who would not normally interact because of being from different disciplines or institutions:

‘I found that … if I had problems with my supervisor, it was easy for me to talk to my mentor, but now if your mentor is your supervisor and you are having problems with the supervisory part then whom are you going to talk to? And you find you’re stuck.’ (Mentor from Makerere University [*sex and age of participant unspecified*])^13^

Online activities via a WhatsApp group formed by one team member have made it possible for team members to spread research mentorship beyond the project. Some WhatsApp group members, although not part of the project have shared testimonies such as positive outcomes after responding to calls shared in the WhatsApp group, and their own experiences including practical tips and the tenacity and persistence required to win such grants:

‘I just want to say, please do apply for calls you see here [*on the WhatsApp platform*]. I got a few regrets but I bagged a generous fellowship when I was just trying out my luck. I’d also like to say take a chance and don’t stop applying each year getting better if you get a regret. I applied five times for another fellowship and got it the fifth time.’ (32 years old, female, PhD student)

### Limitations

Although our results suggest that research mentorship is a good strategy for empowering women in STEMM, there were challenges, which included funding constraints, limited mentorship period, competing interests because of work-related demands, inadequate training, a lack of institutionalising, non-integration within existing systems, limited culture of mentorship and, a lack of understanding about mentorship:

‘In order to spread mentorship benefits widely future efforts should be focused by discipline and level e.g. engineering, vet sciences, biomedical sciences, social sciences, pharmacy, agricultural sciences, medicine, undergraduate students, postgraduate students, postdocs etc.’ (56 years old, female, postdoc)

In agreement with a similar project by Aldina et al. and Murray et al.^14,19^ some mentees thought the role of mentors was to mobilise resources; others feared consequences of mistakes because they did not understand the purpose of mentorship, and a few mentees and mentors expected incentives for additional work. Some mentors experienced compassion fatigue as some expectations for resources were high and unrealistic.^14,19^ The participants, however, lauded the efforts to leverage the experience of different internal and external facilitators, for their own growth. The efforts were dampened by dearth of prior exposure to mentorship, the limited time available for the mentorship activities, and local resource constraints to sustain the initiative.

There were limited common outputs because of the multidisciplinary nature of the group. This made it difficult for some mentors and mentees to effectively communicate and be productive. A major recommendation for future similar projects is to match mentors and mentees based on discipline or be focused on specific projects.

The quality of our M&E was hampered by time constraints, as the project administrative team, M&E team and project team members were preoccupied with full-time work commitments. Engaging an M&E specialist would be an ideal solution. Although our results were satisfactory, these must be taken with caution as they might have been confounded by the high quality of candidates that we selected in this pilot, which may have limited the risk of failure because of dropout, poor execution and low endurance.

## Conclusion

Guided by previous similar programmes,^17,19^ the AFRIESEARCHI Zim GIS Mentorship Program measured indicators of success based on the mentee’s satisfaction with their mentoring relationships, perceived professional growth, and outcome measures, such as career growth, article publications and grant applications.^17^ In addition, this programme identified important areas for improvement for example focusing on disciplines, levels or other common groupings, providing funding support for mentors, mentees and facilitators, continuing activities that increase productivity for example writing articles and applying for grants. Taken together, this pilot project serves as both an advocacy tool and an important preliminary model for strengthening institutional research mentorship for individual academicians and institutions, both within and without Zimbabwe.

### Future directions

To address the challenges encountered and further enhance the effectiveness of the research mentorship programme, we propose the following future directions:

- Securing sustainable funding by exploring diverse funding sources, including government grants, private foundations, and corporate sponsorships and establishing partnerships with organisations committed to empowering women in STEMM.
- Institutionalising mentorship by collaborating with participating institutions to integrate mentorship programmes into their existing structures and developing policies and guidelines to support mentorship initiatives.
- Enhancing mentorship training through the provision of comprehensive training for mentors, focusing on effective communication, conflict resolution, and resource mobilisation and offering training for mentees on mentorship expectations, goal-setting and resource utilisation.
- Improving matching processes, for example, implementing a discipline-specific matching process to facilitate effective communication and collaboration between mentors and mentees and considering a project-based matching to focus on specific research objectives.
- Strengthening M&E by engaging a dedicated M&E specialist to ensure high-quality data collection and analysis and developing a more robust M&E framework to assess programme effectiveness and inform future improvements.
- Scaling up and expanding reach, that is including more participants, institutions and disciplines; and exploring opportunities for regional and international collaborations to share best practices and leverage resources.
- Addressing compassion fatigue and burnout by providing mentors with training and resources to manage compassion fatigue and burnout and encouraging mentors to prioritise self-care and seek support when needed.

By addressing these challenges and implementing these future directions, we aim to enhance the effectiveness and sustainability of our research mentorship programme, ultimately empowering more women in STEMM to achieve their full potential.

## References

[1] Kpokiri EE, McDonald K, Gebreyohannes JA, et al. Research mentorship in low and middle-income countries: A global qualitative evidence synthesis of data from a crowdsourcing open call and scoping review. BioMedRxiv. 2022. 10.1101/2022.09.19.22280121 PMC10773352 38184299

[2] World Economic Forum. The global gender gap report 2020 and 2022 [cited 2023 Aug 23]. Nairobi, Kenya: Women Rights Advocacy (CWRA) and Tenery Research.

[3] Oyebanji O, Okereke E. Empowering African women leaders is key for health equity. Nat Hum Behav. 2023;7:839–841. 10.1038/s41562-023-01603-y 37127793

[4] Dhliwayo B. Women, girls in STEM crucial [homepage on the Internet]. Harare: The Herald; 2019 [cited 2023 Aug 23]. Available from: https://www.pressreader.com/zimbabwe/the-herald-zimbabwe/20191205/281590947433858

[5] Garwe E. Obstacles to research and publication in Zimbabwean higher education institutions: A case study of the research and intellectual expo. Int Res in Educ. 2015;3:10.

[6] The National Academies of Science. USA, partnerships for enhanced engagement in research [homepage on the Internet]. Women in Science Mentoring Program; 2015 [cited 2023 Sep 12]. Available from: https://sites.nationalacademies.org/PGA/PEER/PGA_184663

[7] Bothwell E. Half of academics in Africa receive no research funding. Times Higher Education News. 2018. Available from: https://www.timeshighereducation.com/news/half-academics-africa-receive-no-research-funding

[8] Tagwira F. Key note address by the Permanent Secretary for Higher and Tertiary Education, science and technology development. To the 2018 Student Academic Freedom Regional Advocacy Meeting at Africa University in Mutare, Zimbabwe [homepage on the Internet] [cited 2023 Sep 27]. Available from: https://Safrap.Files.Wordpress.Com/2018/12/2018-Safram-Key-Note.Pdf

[9] Muzira DR, Bondai BM. Perception of educators towards the adoption of education 5.0: A case of a State University in Zimbabwe. East Afr J Educ Soc Sci. 2020;1(2):43–53. 10.46606/eajess2020v01i02.0020

[10] Shuler H, Cazares V, Marshall A, et al. Intentional mentoring: Maximizing the impact of underrepresented future scientists in the 21st century. Pathog Dis. 2021;79(6):ftab038. 10.1093/femspd/ftab038 34283236 PMC8326955

[11] Sachiti R. Mentoring young women in science critical [homepage on the Internet]. Harare: The Herald; 2021 [cited 2023 Oct 02]. Available from: https://www.herald.co.zw/mentoring-young-women-in-science-critical/

[12] UNESCO. Countries urged to invest more in women and girls in science. Harare: UNESCO News; 2020.

[13] Ssemata AS, Gladding S, John CC, Sarah Kiguli S. Developing mentorship in a resource-limited context: A qualitative research study of the experiences and perceptions of the Makerere University student and faculty mentorship programme. BMC Med Educ. 2017;17:123. 10.1186/s12909-017-0962-814. 28709464 PMC5513376

[14] Alidina S, Sydlowski MM, Ahearn O, et al. Implementing surgical mentorship in a resource-constrained context: A mixed methods assessment of the experiences of mentees, mentors, and leaders, and lessons learned. BMC Med Educ. 2022;22:653. 10.1186/s12909-022-03691-2 36045356 PMC9434847

[15] Zhou DT, Maponga CC, Madhombiro M, et al. Mentored postdoctoral training in Zimbabwe: A report on a successful collaborative effort. J Public Health Afr. 2019;10(2):a909. 10.4081/jphia.2019.1081 PMC7118437 32257079

[16] National Academies of Sciences, Engineering, and Medicine. The science of effective mentorship in STEMM. Washington, DC: The National Academies Press: 2019. 31958221

[17] Spence JP, Buddenbaum JL, Bice PJ, Welch JL, Carroll AE. Independent investigator incubator (I3): A comprehensive mentorship programme to jumpstart productive research careers for junior faculty. BMC Med Educ. 2018;18:186. 10.1186/s12909-018-1290-3 30081899 PMC6080403

[18] University at Buffalo. Clinical and translational science institute, Mentor-Protege Mentoring Workshop Series, 2021–2022.

[19] Murray SA, Shuler H, Spencer EC, Hinton A Jr. Mentoring future science leaders to thrive. Trends Pharmacol Sci. 2022;43(6):457–460. 10.1016/j.tips.2022.03.012 35469690 PMC9355866

